# Fast High Resolution Volume Carving for 3D Plant Shoot Reconstruction

**DOI:** 10.3389/fpls.2017.01680

**Published:** 2017-09-28

**Authors:** Hanno Scharr, Christoph Briese, Patrick Embgenbroich, Andreas Fischbach, Fabio Fiorani, Mark Müller-Linow

**Affiliations:** Institute of Bio- and Geosciences, IBG-2: Plant Sciences, Forschungszentrum Jülich GmbH, Jülich, Germany

**Keywords:** image processing, 3D from silhouettes, visual hull, octree, integral image, refinement strategy, performance analysis

## Abstract

Volume carving is a well established method for visual hull reconstruction and has been successfully applied in plant phenotyping, especially for 3d reconstruction of small plants and seeds. When imaging larger plants at still relatively high spatial resolution (≤1 mm), well known implementations become slow or have prohibitively large memory needs. Here we present and evaluate a computationally efficient algorithm for volume carving, allowing e.g., 3D reconstruction of plant shoots. It combines a well-known multi-grid representation called “Octree” with an efficient image region integration scheme called “Integral image.” Speedup with respect to less efficient octree implementations is about 2 orders of magnitude, due to the introduced refinement strategy “Mark and refine.” Speedup is about a factor 1.6 compared to a highly optimized GPU implementation using equidistant voxel grids, even without using any parallelization. We demonstrate the application of this method for trait derivation of banana and maize plants.

## 1. Introduction

Complementary to genomics, the quantitative description of plant phenotypes is at the core of basic research for the analysis of plant development and physiological responses to abiotic and biotic challenges as well as for applications in plant genetic improvement and precision agriculture. An increasing amount of phenotypic data are generated using digital images and time series experiments using a variety of methods and sensors both in controlled environment and in the field (reviewed e.g., in Furbank and Tester, 2011; Fiorani and Schurr, 2013; Mulla, 2013; Araus and Cairns, 2014). Most of these methodologies, ranging from RGB to spectral imaging, are based in high-throughput phenotyping pipelines primarily on 2D spatial analyses for the estimation from image analysis of plant traits such as total leaf area, crop coverage and leaf biomass (Homolova et al., 2013; Chen et al., 2014), leaf color (Hu et al., 2013), plant height measurements using light courtains (Fanourakis et al., 2014) or root morphological features (Das et al., 2015). The use of optical imaging with RGB cameras for estimation of shoot area and above-ground biomass is an established method deployed in indoor phenotyping platforms imaging stations for large-scale studies (Al-Tamimi et al., 2016). These methodologies are based on estimation of leaf area and leaf biomass by acquisition and image analysis of a series of RGB projections from multiple view-angles (Golzarian et al., 2011). Issues that may arise from this approach that generally use a limited number of view-angles are related to the likely underestimation of total leaf area for relatively complex shoot architecture and for large plants at advanced developmental stages. In many cases and for the same reasons, quantifying the growth rates and leaf angles of individual leaves from global shoot images remains challenging. Optical 3D reconstruction of plant shoots using a variety of methods assists in alleviating these issues. For example, studies were conducted in Arabidopsis using light-field cameras for depth reconstruction of rosettes (Apelt et al., 2015), in tobacco using high-resolution 3D imaging and a mesh approach (Paproki et al., 2012), and also in the field using stereo imaging with consumer cameras (Müller-Linow et al., 2015). Progress in this field is still limited by the required computational power and time investment for image analysis (Minervini et al., 2015). In this respect, improvements are required both for methods using 3D reconstruction from silhouettes and 3D imaging.

### 1.1. Related work

Measuring plant geometry from single view-point 2D images often suffers from insufficient information, especially when plant organs occlude each other (self-occlusion). In order to achieve more detailed information and recover the plants 3D geometric structure volume carving is a well established method to generate 3D point clouds of plant shoots (Koenderink et al., 2009; Golbach et al., 2015; Klodt and Cremers, 2015), seeds (Roussel et al., 2015, 2016; Jahnke et al., 2016), and roots (Clark et al., 2011; Zheng et al., 2011; Topp et al., 2013). Volume carving can be applied in high-throughput scenarios (Golbach et al., 2015): For the reconstruction of relatively simple plant structures like tomato seedlings image reconstruction takes ~25–60 ms, based on a well though out camera geometry using 10 cameras and a suitably low voxel resolution 240 × 240 × 300 voxels at 0.25 mm voxel width. Short reconstruction times are achieved by precomputing voxel to pixel projections for each of the fully calibrated cameras. However, precomputing lookup-tables is not feasible for high voxel resolutions due to storage restrictions (Ladikos et al., 2008). Current implementations popular in plant sciences suffer from high computational complexity, when voxel resolutions are high. We therefore implemented and tested a fast and reliable volume carving algorithm based on octrees (cmp. Klodt and Cremers, 2015) and integral images (cmp. Veksler, 2003), and investigate different refinement strategies. This work summarizes and extends our findings presented in Embgenbroich (2015).

Visual hull reconstruction via volume carving is a well-known shape-from-silhouette technique (Martin and Aggarwal, 1983; Potmesil, 1987; Laurentini, 1994) and found many applications. Also octree as multigrid approach and integral image for reliable and fast foreground testing have been used successfully with volume carving in medical applications (Ladikos et al., 2008) and human pose reconstruction (Kanaujia et al., 2013). Realtime applications at 512^3^ voxel resolution have been achieved where suitable caching strategies on GPUs can be applied e.g., for video conferencing (Waizenegger et al., 2009). Here we demonstrate that even higher spatial resolutions are achievable on consumer computer hardware without prohibitively large computational cost. Subsequent octree-voxel-based processing allows extraction of plant structural features suitable for plant phenotypic trait extraction.

## 2. Reconstructing shapes from silhouettes

### 2.1. Voxelbased volume carving

Here, we revisit voxel-based volume carving, closely following the description found in Roussel et al. (2016). In the subsequent sections we will then extend this formulation using octrees as multigrid extension.

Consider an imaging setup with a set of fully calibrated cameras, and a turn-table allowing to rotate our plant of interest around its vertical axis. Golbach et al. (2015) use 10 cameras and no turn-table, Roussel et al. (2016) a single camera and a robot instead of a turn-table; here we use different setups combining up to three cameras and a turntable (see section 3.1).

For each camera *c* we obtain the 3 × 3 intrinsic camera matrix **K**_*c*_, 3 × 3 rotation matrix *R*_*c*_ and translation vector ${\vec{t}}_{c}$ with respect to the reference camera (cmp.Hartley and Zisserman, 2004), and the distance between the origin of our working volume and the reference camera center from calibration (cmp. section 3.2). The origin of the working volume is selected to be the rotation center of the turn-table (cmp. section 3.1).

We acquire *N* images, showing the plant of interest under (equidistantly spaced) rotation angles α_*i*_ where *i* ∈ {1, …, *N*}. We segment each image into a binary mask **M**_*i*_ being one at the foreground, i.e., plant, and zero at background locations. For segmentation, we either use HSV color thresholding (Walter et al., 2007) or a support vector machine (SVM) based learning algorithm (cmp. e.g., Hearst et al., 1998; Wang et al., 2011; Li et al., 2013). Both methods are parameterized offline, where SVM parameters are learned using suitable training data. HSV parameters are hand-tuned by setting 6 threshold values on the 3 color channels. Computational effort as a preprocessing step for carving is negligible, as it typically takes fractions of a second per image for both methods. Subsequently, small objects like noise are removed and small holes filled. Suitable filling sizes depend on the imaging setup and need to be determined empirically.

For each image and thus segmentation mask we calculate the homogeneous camera projection matrix **P**_*i*_, from the rotation angle α_*i*_ by

(1) $$P_{i}=K_{c}\left(R_{c}|{\vec{t}}_{c}+{\vec{t}}_{0}\right)\left(\begin{array}{cc} R_{i} & 0\\ 0 & 1 \end{array}\right)$$

where **R**_*i*_ is the rotation matrix corresponding to the given angle α_*i*_, and translation vector ${\vec{t}}_{0}$ is calculated using the distance of the world origin to the reference camera center, also known from calibration (see e.g., Hartley and Zisserman, 2004). By this, the world coordinate frame rotates with the object, i.e., the plant.

We define an equidistantly spaced, cubic voxel grid around the world origin, being large enough to contain the plant. Such a working volume depends on the plant size in order to keep voxel number and thus complexity low. We can relax this requirement later, when using octrees.

Each voxel center with homogeneous world coordinates $\vec{X}$ is projected to a point ${\vec{x}}_{i}$ in each mask **M**_*i*_ by

(2) $${\vec{x}}_{i}=P_{i}\vec{X}$$

If $\vec{X}$ is projected to the background region of at least one of the *N* masks **M**_*i*_, then this voxel does not belong to the foreground object and its value $\text{V}\left(\vec{X}\right)$ is set to 0, i.e.,

(3) $$V(\vec{X})=\prod_{i=1}^{N}M_{i}({\vec{x}}_{i})$$

Thus, if a voxel belongs to the foreground object, its value $\text{V}\left(\vec{X}\right)$ is set to 1.

When high voxel resolution is desired, and thus runtimes increase, parallelization of the carving algorithm (Ladikos et al., 2008; Brenscheidt, 2014) is feasible. However, as complexity increases linearly with the voxel number and thus cubically with voxel resolution, equidistant voxel discretization quickly reaches its limits for any computer hardware. High resolutions become available on current desktop computer hardware, when hierarchically representing the voxel grid, e.g., as an octree as described next (Szeliski, 1993; Ladikos et al., 2008; Klodt and Cremers, 2015).

### 2.2. Octrees

**Figure 1:**
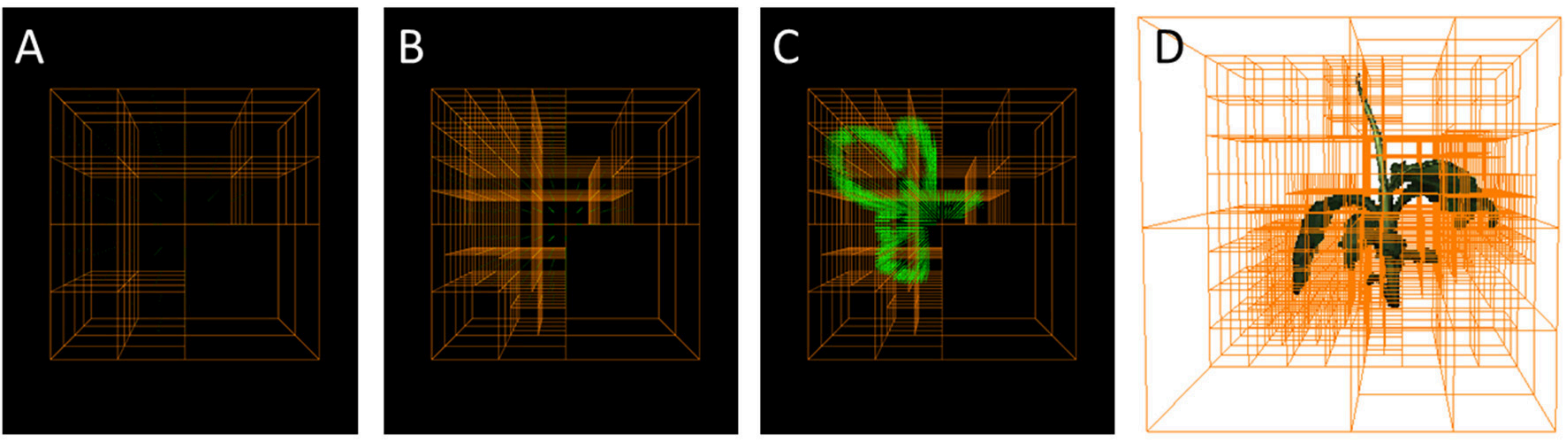
Octrees: A well-established multigrid data structure reducing complexity wrt. an equidistantly spaced voxel grid. **(A–C)** Selected refinement steps in an Octree-based reconstruction. Foreground corner points are green. **(D)** Reconstructed plant with corresponding Octree.

An *Octree* (Meagher, 1980, 1995; Szeliski, 1993) is a hierarchical tree data-structure. Each node corresponds to a cube in 3d space (i.e., a large or small “voxel”) where each node not being an end-node or “leaf” is subdivided in eight child nodes. The child nodes have each half the size, i.e., edge length, of their parent node and are non-overlapping such that they fill the same volume as their parent. (cmp. Figure 1). Octrees are designed to efficiently store voxel grids, where large, unstructured volumes can be stored using only few nodes (i.e., large voxels), whereas small structures or surfaces in space can be finely resolved using nodes further up in the tree (i.e., small voxels), i.e., higher “level” nodes. To this end, when building up a representation of an object, an octree node is kept as an end-node, when the corresponding 3d volume is either empty, or completely filled by the object. When the corresponding volume is only partly filled by the object, the node is split into its eight children. This is iterated until the finest resolution is reached. Then leaf nodes at finest resolution can be considered as partly filled and represent surface voxels of the object.

Consequently, memory investment for storing a working volume only marginally depends on its size, but rather on the size and complexity of the object of interest. For example storing an equidistantly sampled working volume of 1 m^3^ using voxels of 0.25 mm edge length requires 6.4·10^10^ voxels or ~64 GB when representing each voxel by a single byte without any compression. Storing the same working volume takes only a few bytes, when it is empty, and when occupied by an object, still much less than storing an equidistantly sampled version of the occupied volume only (see section 4.1 for an example). We can therefore select our initial working volume such that it well contains the overall visible volume of our setup, without compromising on runtime.

### 2.3. Integral image

**Figure 2:**
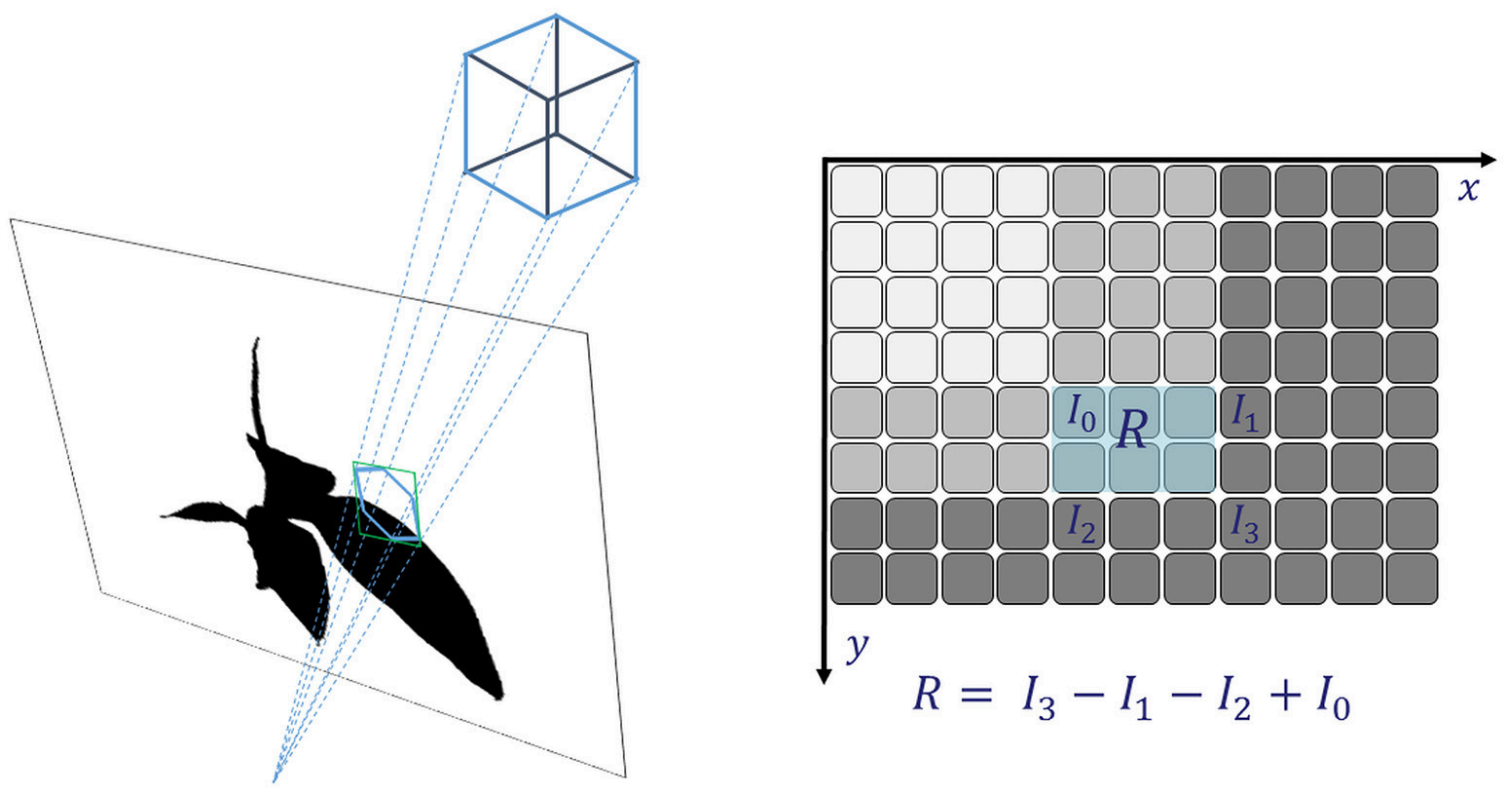
Integral images: fast and reliable checking if a (potentially large) voxel covers fore- and background and thus needs to be refined.

The occupancy test in Equation (3) assumes, that a each voxel $\vec{X}$ corresponds to a single pixel or point ${\vec{x}}_{i}$ in a mask image **M**_*i*_. This can be sufficiently well fulfilled when image resolution is low, such that pixel size at working distance is well above voxel size. For large voxels as in the lower levels of an octree, this assumption is not sufficiently fulfilled. We need to adapt the occupancy test, such that we can decide if a voxel is empty, completely or only partly filled. Consider a large voxel being projected to a mask image (see Figure 2, left). In general, the 2d central projection of a cube is a hexagon and we needed to test every pixel within the region of this hexagon. When foreground is 1 and background 0, this corresponds to integrating the mask image over the region of the hexagon and compare the result *R*_hex_ with the area of the hexagon *A*_hex_. Then only when *R*_hex_/*A*_hex_ is exactly 1 or 0, the octree node would not be split. However, integrating over a hexagon for every voxel is time consuming. Instead of this, we integrate over the bounding-box of the hexagon, yielding *R*_bbx_, as this can be done very efficiently using the integral image approach. A bounding-box is a rectangle with sides parallel to the coordinate axes, being spanned by ${\vec{x}}_{\text{min}}$ as the upper left corner and ${\vec{x}}_{\text{max}}$ as the lower right corner^1^. Here ${\vec{x}}_{\text{min}}=\left(x_{\text{min}},y_{\text{min}}\right)$ contains the minimum coordinate values^2^ of all corner points of the voxel projected to the mask image, and ${\vec{x}}_{\text{max}}$ their maximum^3^, accordingly. In case the bounding-box is completely filled by the object, i.e., *R*_bbx_ = *A*_bbx_ or completely empty, *R*_bbx_ = 0, it will be the same for the hexagon.

(4) $$A_{\text{bbx}}=(x_{\text{max}}-x_{\text{min}})\cdot (y_{\text{max}}-y_{\text{min}})$$

is the area covered by the bounding box. For a partly filled bounding-box we will split the corresponding octree node, in order to be on the safe side, even though there may be cases when the bounding box is partly filled, but the hexagon actually is not.

The integral over the bounding box can be evaluated very efficiently using the summed area table (Crow, 1984) or *Integral Image* **I**_*i*_ of the mask **M**_*i*_ (see Figure 2, right, and Veksler, 2003; Viola and Jones, 2004). Every pixel of **I**_*i*_ contains the integral of **M**_*i*_ in the rectangle being spanned by ${\vec{x}}_{\text{min}}=\vec{0}$ and ${\vec{x}}_{\text{max}}$ being the current pixel.

(5) $$I_{i}(x,y)=\sum_{x\prime \leq x,y\prime \leq y}M_{i}(x\prime ,y\prime )$$

**I**_*i*_ can be derived efficiently by a recursive convolution scheme starting at the upper left corner and proceeding row- or column-wise, where we calculate the current pixel value from the value of its upper and left neighbors, i.e.,

(6) $$I_{i}(x,y)=M_{i}(x,y)+I_{i}(x-1,y)+I_{i}(x,y-1)-I_{i}(x-1,y-1)$$

with considering **I** = 0 outside the mask image. From the integral image the sum over any rectangular region can be calculated using four values

(7) $$\begin{array}{l} R_{\text{bbx},i}=I_{i}(x_{\text{min}},y_{\text{min}})-I_{i}(x_{\text{min}},y_{\text{max}})-I_{i}(x_{\text{max}},y_{\text{min}})\\ \;+\;I_{i}(x_{\text{max}},y_{\text{max}}) \end{array}$$

For border handling, bounding-box corners outside the current field of view, are moved to the nearest pixel in **I**_*i*_.

### 2.4. Volume carving with octrees

When using octrees instead of equidistantly spaced, cubic voxel grids, the occupancy test from Equation (3) needs to be exchanged. To do so, we first calculate for every mask image **M**_*i*_ the corresponding integral image **I**_*i*_ using the scheme from Equation (6).

We start with an octree containing only a single coarse voxel covering the whole working space, i.e., the trunk node of the octree. This node is initially marked as “object,” i.e., it is assumed it contains an object. Then, for all images, all nodes are iteratively updated, when a node *N* is marked as “object” and

(8) $$R_{\text{bbx},i}/A_{\text{bbx},i}=\{\begin{array}{cc} 1 & \text{ then }N\text{ is marked as }“\text{object}”\\ 0 & \text{ then }N\text{ is marked as }“\text{empty}”\\ \text{else} & N\text{ is marked as }“\text{refine}.” \end{array}$$

Voxels marked as “refine” are split, marked as “empty” and their Children are marked as “object.” This update scheme is iterated until the desired finest resolution is reached. In the following, “one iteration” means projecting all current octree voxels on one image once. We have several options, how to organize the iterations:

1. Depth first: On the current image, we iterate until all nodes are split to the finest resolution, where we visit each image only once, or
2. Breadth first: We make a single pass over all nodes for the current image, split the ones marked as “refine,” and go to the next image for the next pass, where we visit each image multiple times.
3. Refining resolution: Set the maximum resolution to a coarse level and iterate breadth first to convergence. Then set the resolution such that finest voxels are allowed to be split once and run breadth first once. Repeat the last step until the desired resolution is reached.
4. Mark and refine: Make a single pass over all nodes for all images, only marking nodes, not actually splitting them. Then split all nodes marked as “refine.” Iterate until the desired resolution is reached.

We investigate memory usage and runtimes for these different refinement strategies in our experiments (see section 4.1).

For our experiments with the banana plants we used a finest resolution of 0.244 mm, being a bit smaller than the pixel size at working distance of ~0.3–0.4 mm depending on the camera distance to the voxel of interest. The volume of the visual hull of a plant is estimated by summing over the volumes of all nodes marked as “object” where nodes at the finest resolution, i.e., with unknown filling percentage, are accounted for half their volume in order to get an unbiased estimate. Clearly, as the visual hull includes convex volumes or other occluded volumes, its volume only coarsely reflects the volume of a complex object such as a plant.

For visualization (cmp. e.g., **Figures 7**, **8**) we assigned to each “object” voxel a single RGB color value for simplicity. To this end, we calculated for each voxel its nearest camera^4^ and selected the color of the center pixel of the voxel's bounding box. No visibility or plausibility tests have been performed. Please note, that this approach is good for visualization purposes, only, and is not well suitable for color-based trait evaluation.

## 3. Materials and methods

### 3.1. Imaging

Two setups for image acquisition were used for this study, a semi-automatic setup combining a turntable and up to two cameras, as well as a fully automated system including a turntable and three industrial cameras.

**Figure 3:**
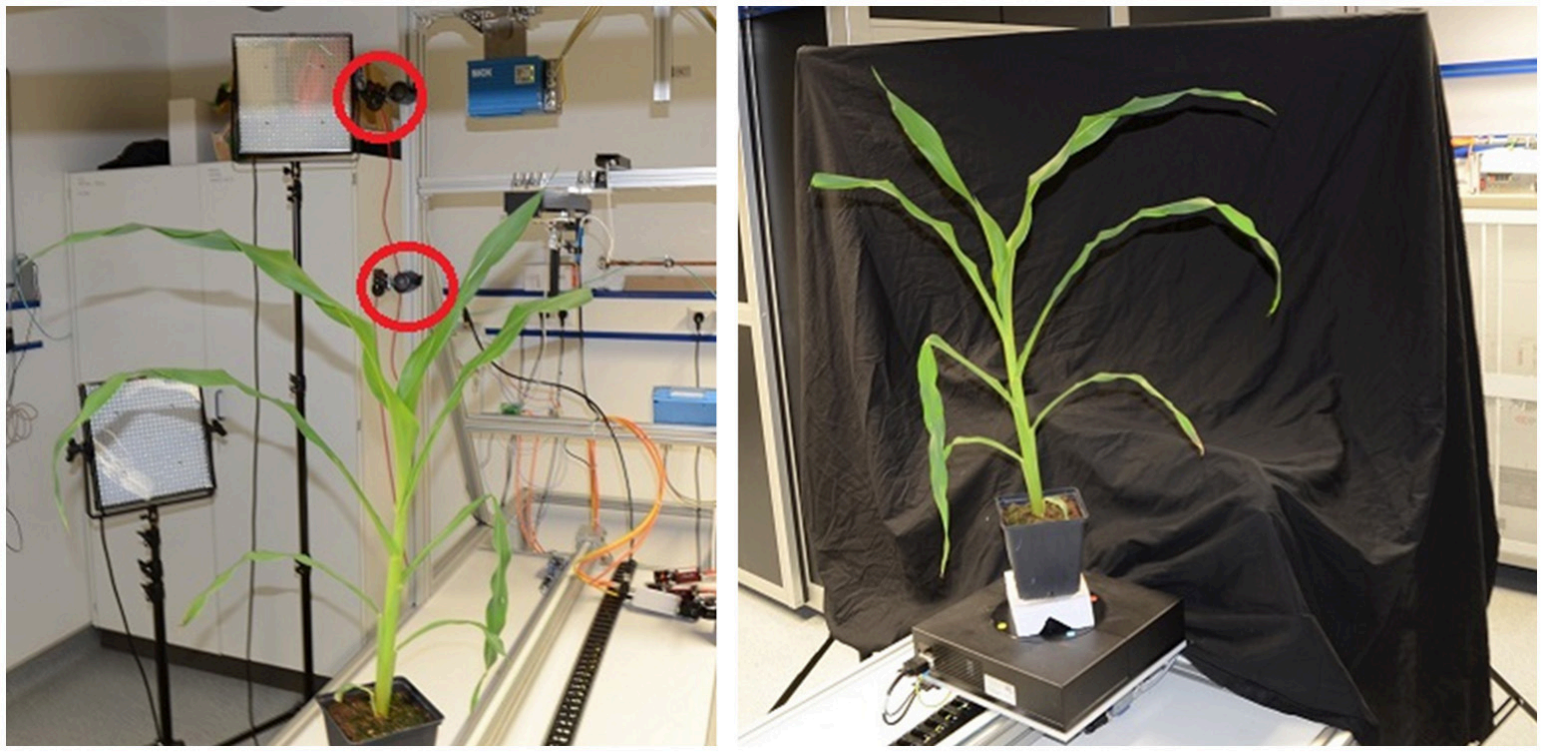
Semi-automatic imaging setup. **(Left)** View from behind the plant with cameras indicated by red circles. **(Right)** Setup as seen from the lower camera.

The semi-automatic setup (see Figure 3) needs manual initial positioning of a plant, which is then imaged fully automatic. This setup includes an automated turntable (Steinmeyer Mechatronic, Dresden Germany, DT180-SM, 0.1° accuracy) with color markers for image-based rotation angle estimation, and two industrial cameras (Allied Vision Technologies, Stadtroda Germany, AVT MANTA G-504, 5MP color camera). Images can be taken either in a stop-and-go fashion, where the plant is turned to the next pose, stopped, and then images are acquired, or alternatively, the turntable can rotate and images are taken in equidistant time intervals corresponding to equidistant rotation angles.

**Figure 4:**
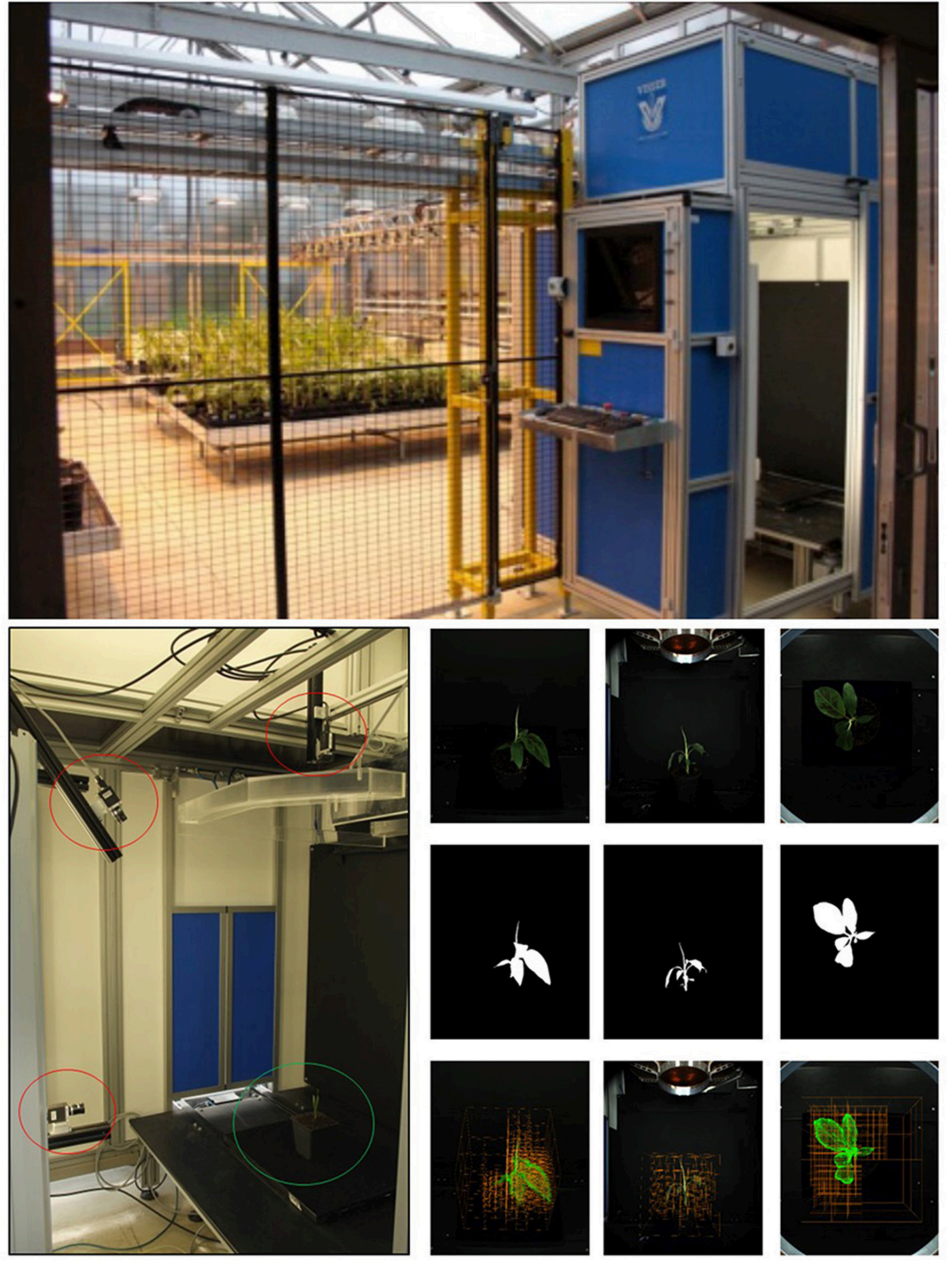
Imaging. **(Top)** Screenhouse setup. **(Bottom left)** Camera setup in Screenhouse measurement chamber. Three 5 MP RGB cameras (red circles) with different view angles and rotating table (green circle). **(Bottom right, top)** Original RGB images taken from 3 different view angles; **(Middle)** Binary masked images; **(Bottom)** Intermediate carving step overlaid on images.

The fully automated setup called *Screenhouse* at IBG-2 is used for screening of the shoot structure and function of different mono- and dicotyledonous plant species in a greenhouse environment (cmp. Nakhforoosh et al., 2016). Plants are automatically fed to the system being equipped with an imaging station for automated imaging (see Figure 4, top and left). Imaging is routinely performed with three cameras (Point Gray Grasshopper2, 5MP color camera, by FLIR Integrated Imaging Solutions Inc., Richmond, British Columbia, Canada) which are located at different positions to efficiently assess the plant structural properties for diverse shoot architectures. A turntable is used for stop-and-go imaging at 4 positions at 0, 90, 180, and 270°. For the top camera only the image acquired at 0° is used. This means that 9 images per plant are used for reconstruction in our experiments.

### 3.2. Camera calibration

One of the main drawbacks of visual hull computation with fixed calibrated cameras is its sensitivity to imprecise external camera calibration and lens aberrations. When a mask **M**_*i*_ is misaligned and thus does not well overlap with the “true” object volume, the non-overlapping parts are deleted from the volume without further testing or corrections. Geometric precision of the calibration needs to be in the same range as the desired precision in object space, i.e., when reconstructing relatively large objects sufficient precision is reachable using well established methods. When dealing with small objects like plant seeds, an online image-based camera pose calibration step may be needed (see e.g., Roussel et al., 2016). In our case, plants are in the size range of tens of centimeters and precision needed for calibration is in the range of ≈0.3 mm, which is approximately the lower limit of pixel size at working distance. We use the calibration approach introduced by Bouguet (1999), where multiple, here 20, images of arbitrarily posed flat calibration targets are used. Laser-printed calibration targets glued to flat material are sufficient for our requirements. We use the OpenCV ver. 2.4.9 (Bradski, 2000) implementation *calibrateCamera* of the method presented in Zhang (2000).

### 3.3. Software implementation

The software framework was implemented in C++ on a Windows 7 operating system with Microsoft Visual Studio 2013.

### 3.4. Datasets acquired

#### 3.4.1. Maize plants on the semi-automatic setup

**Figure 5:**
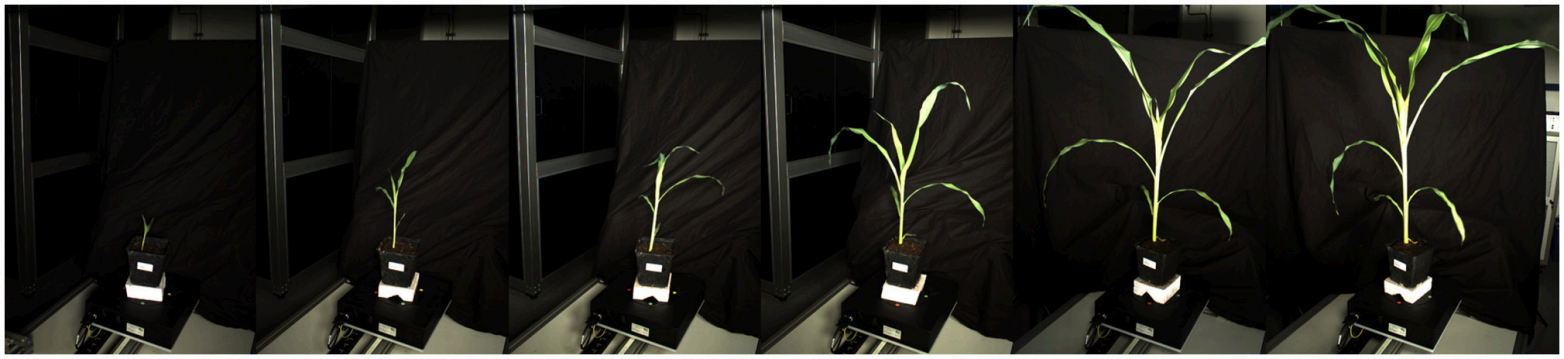
Images of the Maize experiment. The images show the same maize plant over the course of the experiment at roughly the same rotation angle. Imaging was done with continuous rotation and equidistant time intervals for image acquisition using the semi-automatic setup (see section 3.1).

We imaged 5 maize plants (Zea Maize, Genotype “Badischer Gelber”) at 6 different time points over 17 days from seedling stage with 2–8 leaves stage (see Figure 5 for an example). Using both cameras of the semi-automatic setup this resulted in 30 datasets of 60 images each, where we had to remove 7 sets due to imaging problems, e.g., plants being already too large for our setup.

#### 3.4.2. Banana seedlings on the automatic setup

**Figure 6:**
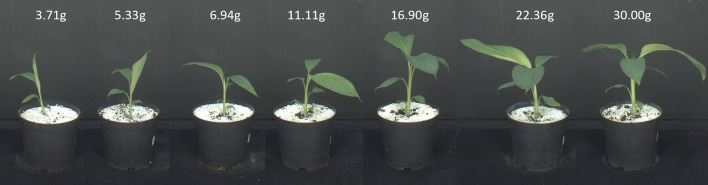
Images of the Banana experiment. The images show one banana seedling of each of the seven groups. Imaging was done using the fully automatic Screenhouse facility (see section 3.1).

**Figure 7:**
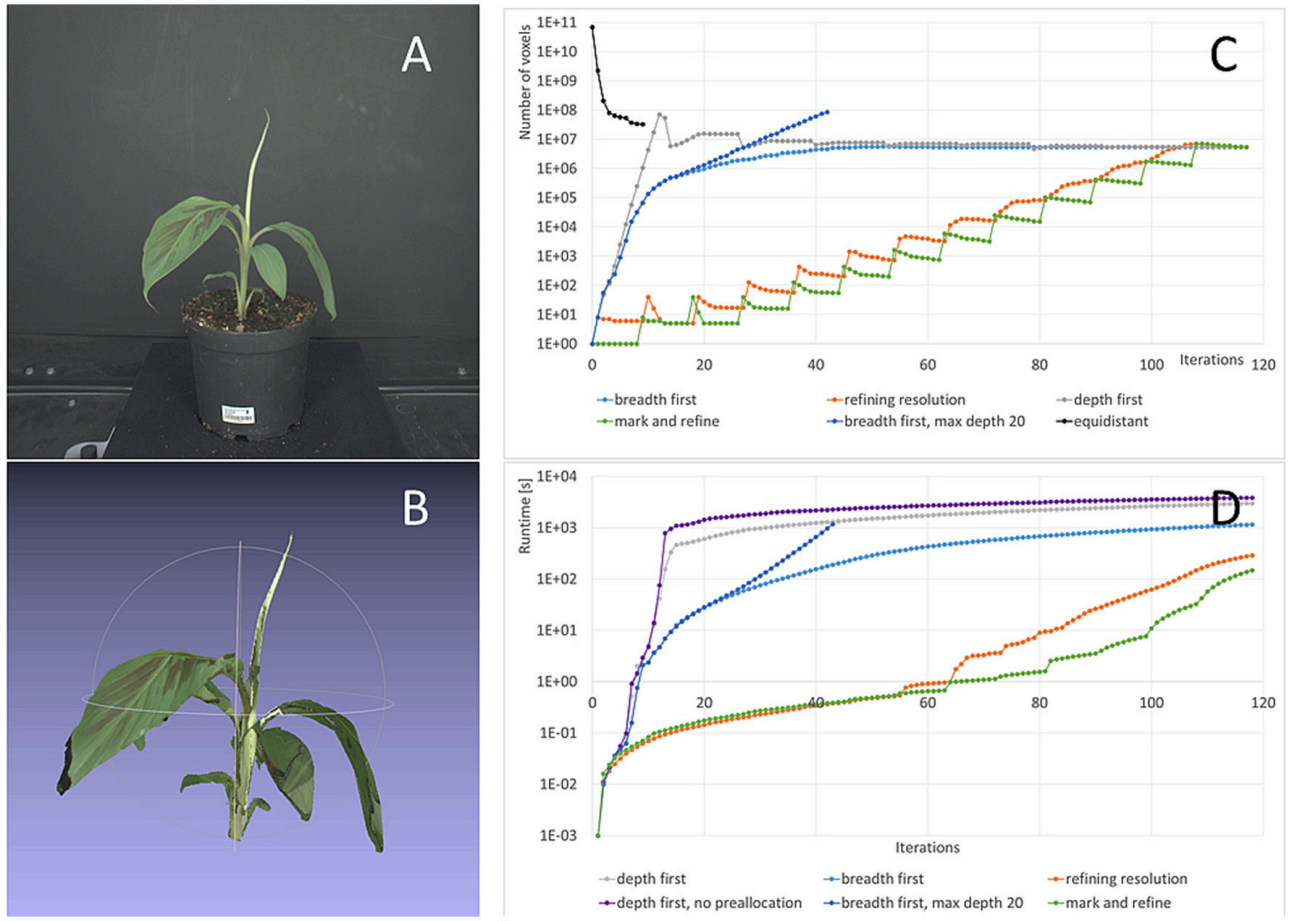
Refinement strategies: Storage complexity and runtimes. **(A)** One original image from the banana dataset used for this test. **(B)** 3d colored point cloud at final resolution (0.244 mm). **(C)** Number of voxels used with our method and the different refinement strategies from Section 2.4 and the classic approach with equidistant sampling. **(D)** Runtimes for different refinement strategies.

Seven groups of five banana seedlings, cultivar Khai Thong Ruang, were imaged and harvested for fresh weight determination immediately after imaging. Imaging and harvesting took place 6, 10, 14, 17, 20, 28, and 34 days after transplantation of the initial four leaf plantlets. This resulted in 35 datasets of 9 images each (see Figure 6). In addition some larger banana seedlings have been imaged in the same way, but not been harvested (see Figure 7).

## 4. Experiments

### 4.1. Performance of different refinement strategies

We investigate the runtimes needed for the four different refinement strategies *Depth first, Breadth first, Refining resolution*, and *Mark and refine* described in Section 2.4. We performed these test on a computer with 48GB RAM and two Intel Xeon E5540 @ 2.53 GHz as some refinement strategies use a lot of memory. The *Mark and refine* strategy can also be run on a current laptop (8GB RAM, Intel Core i5-5300U @ 2.30 Ghz) at comparable speeds. All runtime tests were done without any parallelization using a single CPU only, unless stated differently.

In Figure 7A an image from the dataset used for this investigation is shown, one of the larger banana plants being 31 cm high and 29 cm wide. Figure 7B shows the corresponding carving result. Its final 3d grid contains $N_{oct}=5.4\cdot 10^{6}$ foreground octree voxels from 0.244 to 15.6 mm. We observe that finest structures, like the tip of the top leaf or the tip of the large leaf on the left are lost. Missing of finest structures in volume carving typically, and also here, has two main reasons: segmentation errors and calibration inaccuracies. The main effect in the data set used here is lens imperfections being not fully compensated by radial distortion correction. In addition, the brownish part of the tip of the top leaf is missing in one segmentation mask and therefore carved away. For the purpose of this publication, i.e., performance evaluation in terms of runtime and resolution, such inaccuracies are of no interest. However for practical applications needing such detail, one may want to invest in better calibration procedures and more precise segmentation algorithms.

A uniform 0.244 mm grid requires 6.9·10^10^ voxels to represent the same 1 m^3^ working volume (corresponding to 64 GB, when using 1 byte per voxel), where $N_{equi}=3.3\cdot 10^{7}$ voxels are foreground i.e., plant. We see that while using an equidistant grid during carving requires a large amount of memory, this is not necessarily the case for the final result. In our case the difference is a factor of *N*_*equi*_/*N*_*oct*_ = 6.1, allowing to perform subsequent processing using equidistantly sampled foreground voxels, if needed.

Complexity for deriving this carving result heavily depends on the refinement strategy used. Figure 7C shows the number of octree voxels generated when iterating through the images of the dataset. As before, “one iteration” means projecting all current octree voxels on one image once.

For the classic equidistantly sampled voxel grid approach, we would need 9 iterations as we have 9 images in the banana dataset. We derived the foreground voxel number plotted in Figure 7C from the *Depth first* results using octrees.

For the octree-based method, in order to reach the finest resolution of 0.244 mm, the initial 1 m^3^ voxel needs to be refined 12 times. As some final surface voxels may only be seen from a single image, we have to project the voxels to each image 12 times to make sure to reach the final resolution; and one more time to remove the unneeded (background) voxels at final resolution. I.e., 117 iterations are needed overall.

The first recommendation for speed-up, or to be able to finish at a usable result at all, is that the final octree resolution needs to be restricted to the desired resolution. If this restriction is relaxed, many octree voxels are refined unnecessarily. This can be seen in Figure 7C, case “breadth first, max. depth 20,” where the number of voxels keeps increasing exponentially after 18 iterations, in contrast to the case “breadth first,” where we restricted resolution. The unrestricted calculation leaded to memory overflow and ended before reaching the final result.

The second recommendation for speed-up is to preallocate memory wherever possible. The effect of allocation on the fly^5^ is demonstrated in Figure 7D, cases “depth first” and “depth first, no preallocation” where a factor of 1.3 longer final runtimes can be observed.

Generally the *Depth first* strategy shows by far longest runtimes. The reason is that in the first iterations the number of voxels generated increases exponentially, in our case to 75 M voxels after 12 iterations. This is when the finest resolution is reached for the first image. Then, with every new image voxel number drops with the first iteration, as many voxel s are carved away, and then again increases with the next iterations. Due to the large number of voxels to process, runtime is very high (2,997 s).

The *Breadth first* strategy is about a factor 2.6 faster needing 1,155 s to finish. Restricting the reachable resolution with every loop through all images, i.e., using the Refining resolution strategy again yields a speed-up of a factor 4.0, i.e., a factor of 10.4 compared to *Depth first*.

*Mark and refine* is the fastest refinement strategy, as it produces fewest voxels during processing. It reaches a resolution of 0.98 mm after 7.7 s ($\hat{=}1,024^{3}$ voxel grid), 0.49 mm after 32.7 s ($\hat{=}2,048^{3}$ voxel grid), and the final 0.244 mm after 148 s ($\hat{=}4,096^{3}$ voxel grid). Using *Mark and refine* on the laptop described above the final result is reached after 85.7 s, i.e., a factor 1.7 faster. In Roussel et al. (2016) 218 s are reported for the usual equidistant grid method using a 1, 024^3^ voxel grid and a CPU implementation, 12.56 s using a well optimized GPU implementation. We even outperform this GPU optimized version by a factor 1.6, using no parallelization at all.

We also tested a simple parallelization method for *Mark and refine*, by distributing the voxel-to-image projections over 8 CPU threads, but not threading the splitting step. The final result at 0.244 mm resolution is then reached after 37.7 s. Compared to the slowest (but still completing) method we observe an overall speedup of a factor 102.

### 4.2. Runtime for different plant sizes

The overall runtime of octree-based volume carving strongly depends on the plant size and complexity, more precisely, the surface area of its visual hull, as the finest voxels occur there. Consequently, we observe that the number of pixels roughly increases by a factor of 4 after each pass through all images in *Mark and refine* (cmp. Figure 7C). This is consistent with the assumption that on average 4 of 8 of the new voxels are foreground, when the old voxel was a the surface and thus needed splitting.

**Figure 8:**
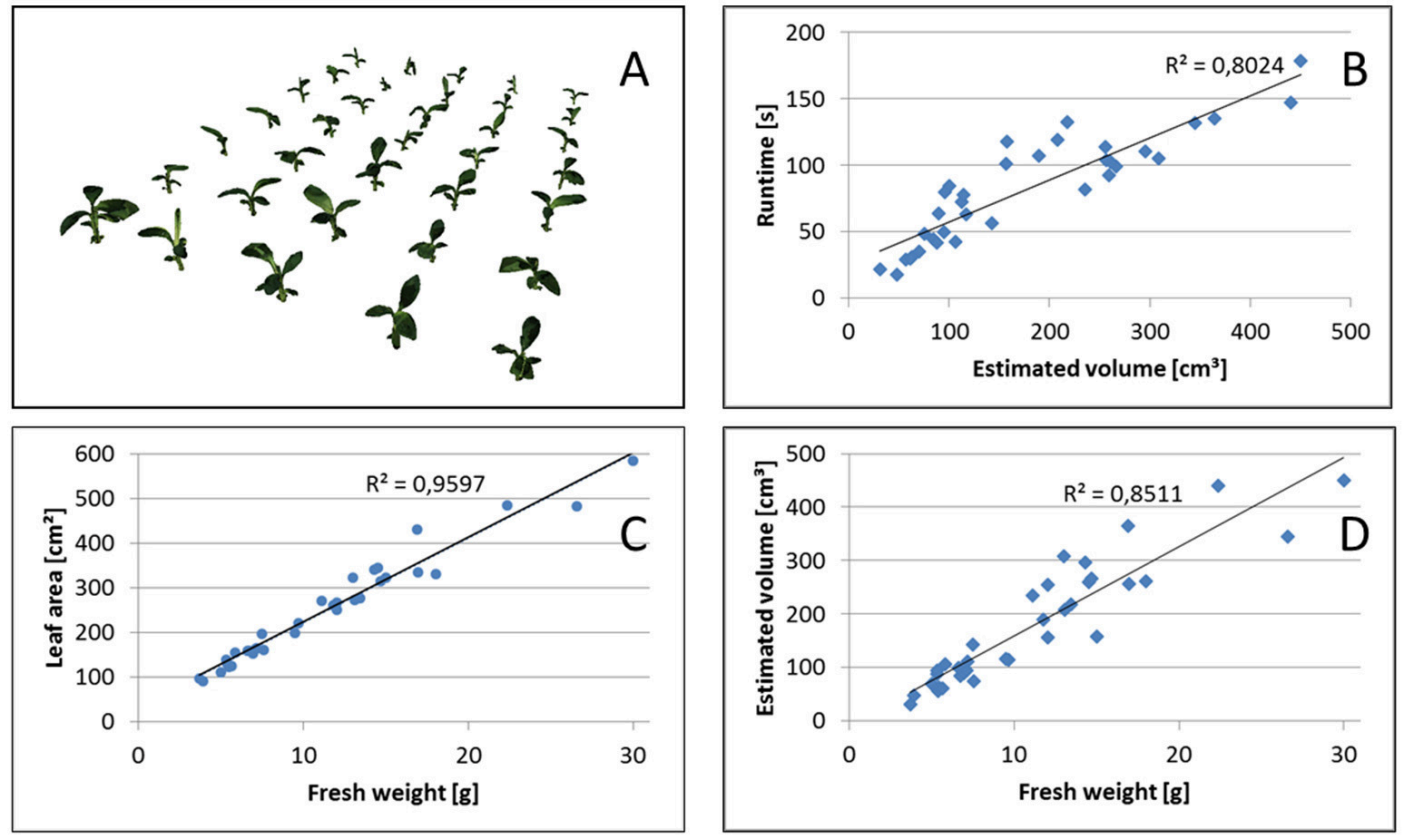
Volume estimation. **(A)** Reconstructed set of 35 banana plants. Correlation between **(B)** Runtime and estimated volume, **(C)** leaf area and fresh weight, as well as **(D)** estimated volume and fresh weight.

Overall runtimes for the reconstruction of the 35 plants of the Banana datasets are shown in Figure 8B. We observe, that runtimes are faster for smaller plants, approximately linearly increasing with the estimated volume or, for banana plants, with leaf area. This is the case as the structure of banana plants is dominated by big leaves, i.e., flat objects with area being well correlated with volume or, equivalently, fresh weight (see Figure 8C).

### 4.3. Examples for visual-hull based plant traits

We evaluated the general usability of this visual hull reconstruction approach with two scenarios using banana and maize plants (see section 3.4).

In Figure 8A reconstructions of the banana plants are visualized and in Figure 8D their fresh weight is plotted vs. the estimated volume. We observe that the estimated volume of the visual hull correlates with the fresh weight (*R*^2^ = 0.8511), however differences in estimated volume for plants with approximately the same weight are up to about a factor of 2. This makes the estimated volume useful as non-invasive surrogate measure for fresh weight in statistical analyses with replication, but not very reliable when comparing individual plants. This is not unexpected, as the visual hull may contain non-negligible non-plant volumes due to occlusions. Such non-plant volumes largely depend on the actual configuration of leaves, i.e., leaf poses.

**Figure 9:**
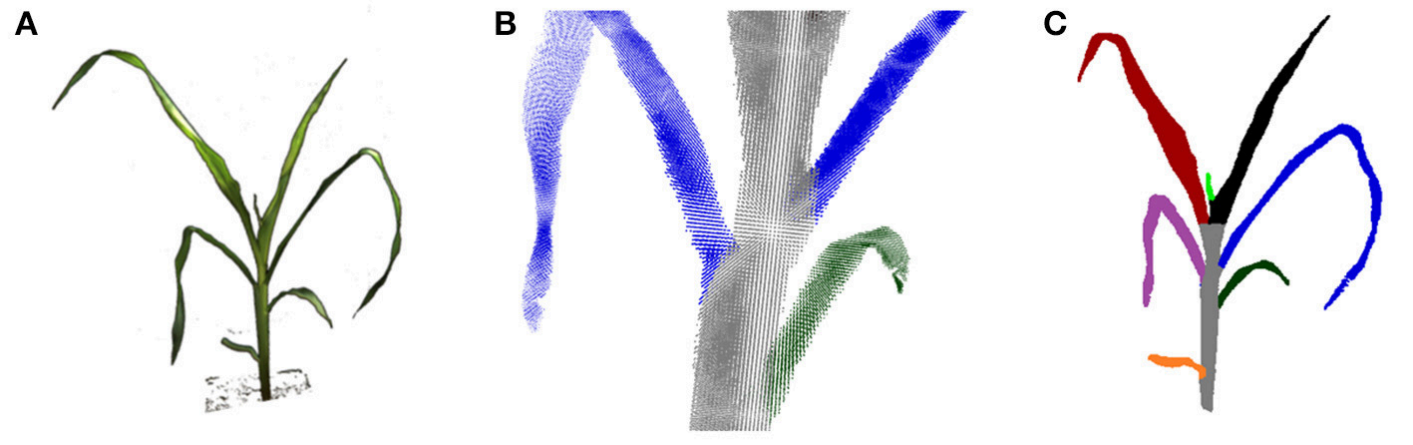
Leaf and stem segmentation for maize. **(A)** An input image (foreground only). **(B)** Close up: leaf-stem segmentation in the point cloud. **(C)** Segmented plant.

The visual hull can be used more reliably to derive structural plant properties, like leaf numbers or leaf areas, when applying further processing. In Figure 9 a leaf segmentation result is depicted for a maize plant. Here a reliable leaf-wise segmentation has been reached by a sequential cluster connecting algorithm and subsequent refinement steps. A detailed description of the algorithm is beyond the scope of this publication. In short, leaf segmentation is done by searching clusters in horizontal one final voxel thick slices, and connecting the found clusters over slices scanning from top to bottom. By this, cluster labels are transferred from top to bottom, forming plant parts. When two clusters merge in one slice, the smaller label is transferred to this slice cluster. By this strategy leaves are segmented, where the stem region still belongs to the top-most leaf. They are separated in a subsequent step using simple but heuristic geometry rules. This leaf segmentation allowed e.g., to count leaves reliably. For 19 of the 23 maize plant datasets leaf numbers were correctly estimated. For the other 4 plants the smallest top leaf was missed, comparable to the small bright green leaf in Figure 9C. For further details we refer to Embgenbroich (2015).

**Figure 10:**
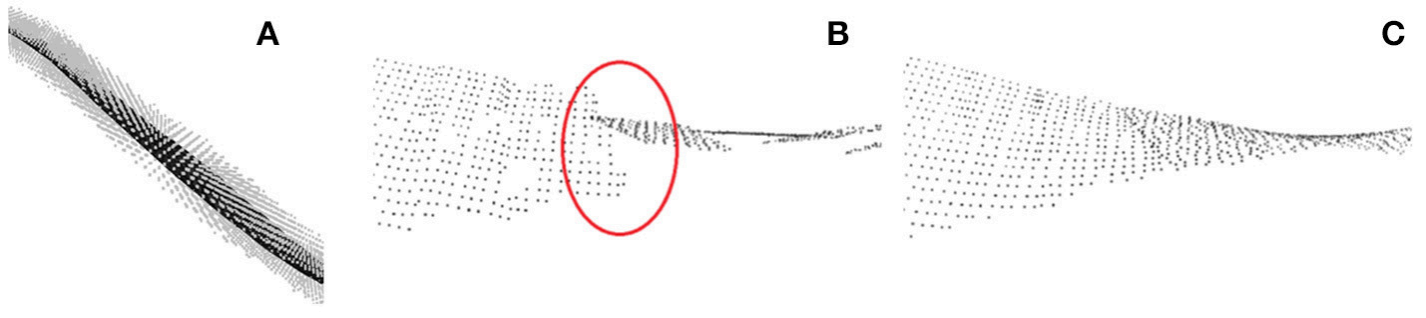
Leaf area estimation. **(A)** Plane fit through local leaf point cloud. **(B)** Piece-wise plane fits with discontinuities. **(C)** Smooth surface via moving least squares fit.

When individual leaves are segmented as point clouds, leaf properties can be derived. The length of a leaf may be determined as the shortest path through the point cloud connecting leaf base and tip. To do so, usual skeletonization algorithms may be used. In order to derive leaf area, the point cloud needs to be flattened. This can be done by piece-wise fitting planes to the leaves as depicted in Figure 10. In order to remove steps between fitted plane pieces (Figure 10B), a moving least squares method may be applied (Figure 10C). For further details we refer to Embgenbroich (2015).

## 5. Conclusion and outlook

The presented volume carving using octrees and integral images allows fast and accurate visual hull computation on standard PC hardware with relatively low memory and CPU requirements. A key to efficient computations is the choice of a suitable refinement strategy, as unrestricted refinement leads to exponential “explosion” of voxel numbers and thus complexity. Speedup between efficient and non-efficient implementations can be more than 2 orders of magnitude and differences in memory requirements comparably dramatic. Efficient CPU implementations allow fast execution on usual laptop or desktop compute hardware, outperforming even GPU-optimized “brute force” implementations using equidistantly sampled voxel grids. Even faster implementations may be possible, when porting the presented multi-grid approach to GPUs. Usability of volume carving results represented in octree voxel grids is equivalent to using fine equidistant voxel grids, but at much higher reachable resolutions. Some examples how to derive traits of banana and maize plants have been presented to demonstrate possible applications in plant phenotyping for deriving leaf level traits.

## Author contributions

FF, MM, and HS designed the study. PE, CB, AF, and HS implemented and tested the software. CB and PE performed all lab experiments. FF, MM, and HS drafted the manuscript. All authors contributed to text and figures of the manuscript and approved the final manuscript.

### Conflict of interest statement

The authors declare that the research was conducted in the absence of any commercial or financial relationships that could be construed as a potential conflict of interest. The reviewer SL and handling Editor declared their shared affiliation.

## Footnotes

^1^The image coordinate origin is assumed to be in the upper left corner and *x*- and *y*-axes pointing to the right and downwards, as usual for images.

^2^Rounded to lower values, using the C++ operation *floor*.

^3^Rounded to higher values, using the C++ operation *ceil*.

^4^We used as camera position the camera center, as usual.

^5^Such allocation is as offered e.g., in C++ using the vector-class from the standard template library.
